# Do statins reduce the rate of revision surgery after chronic subdural hematoma drain?

**DOI:** 10.1007/s00701-021-04871-7

**Published:** 2021-05-25

**Authors:** Johann Klein, Lisa Mauck, Gabriele Schackert, Thomas Pinzer

**Affiliations:** Department of Neurosurgery, University Hospital Carl Gustav Carus, Technische Universität Dresden, Fetscherstrasse 74, 01307 Dresden, Germany

**Keywords:** Chronic subdural hematoma, Statins, Burr hole craniotomy, Hematoma drain

## Abstract

**Background:**

With chronic subdural hematoma (CSDH), surgery is the therapeutic mainstay for large or symptomatic cases. Statins are reported to be effective as the primary therapy of CSDH to obviate the need for surgery. However, the effect of statins on the postoperative course of CSDH is largely unclear. We therefore sought to determine whether statins reduce the rate of repeat surgery after CSDH drain.

**Methods:**

We performed an analysis of all patients who underwent surgery for CSDH at our institution between 2012 and 2018. The patients were separated into those who received statins as part of their previous medication (statin group) and those who did not (control group). The medical records were reviewed for repeat surgeries and complications. Additionally, patients or their relatives were contacted via phone to obtain missing data and inquire about possible repeat surgeries at other institutions.

**Results:**

We identified 407 patients who received CSDH evacuation via burr hole craniotomy. In total, 123 patients were treated with statins as part of their daily medication. Repeat surgery was performed in 26 patients in the statin group (21.1%) and 57 patients in the non-statin group (20.1%, *p* = 0.81). Upon multivariate logistic regression analysis, neither of the variables statins, age, antithrombotic medication, Charlson comorbidity index, or Markwalder grading score yielded a statistically significant effect upon the revision rate.

**Conclusions:**

We found no evidence for the protective effect of statins in patients who underwent surgery for CSDH. We thus conclude that statin therapy is not warranted for CSDH perioperatively.

## Introduction

Chronic subdural hematoma (CSDH) is a common pathology in neurosurgical practice with an increasing incidence as the population grows older[16]. With large or symptomatic hematoma, surgical evacuation and placement of a subdural drain are the therapeutic standards [14, 18]. Conservative treatments are increasingly researched to obviate the need for surgery but the overall evidence remains sparse [19].

Statins are established drugs in the management of blood cholesterol and in the prevention of cardiovascular events [3, 17, 28]. Moreover, they are favored by some authors as a promising treatment for CSDH, and a randomized controlled trial showed a more pronounced reduction in hematoma volume after 8 weeks of treatment with atorvastatin as compared to placebo [7]. However, despite the growing interest in the treatment of CSDH with statins, they have been poorly researched as an adjunct to surgical hematoma evacuation. Therefore, we performed a study to learn whether patients who were administered statins had a lower rate of repeat surgery after the initial evacuation of CSDH compared to patients who did not receive statins. As secondary outcome parameters, we sought to determine the reasons for revisions—insufficient hematoma resolution, recurrent hematoma, acute postoperative hematoma, or wound healing disturbance—and whether other factors such as the use of antithrombotic medication or the patients’ age could be established as risk factors.

## Methods and materials

The study protocol was approved by the institutional review board (EK 37022017). We retrospectively searched our database for all patients who underwent surgery for CSDH between 2012 and 2018. Patients were excluded if they had pure hygroma, a congenital anomaly of the central nervous system, if they had undergone a craniotomy before, if the CSDH was a result of a CSF leak, or if they died of an unrelated cause within 1 month of the surgery, as the latter may not have allowed for a sufficient follow-up time to detect recurrent CSDH.

The surgeries were performed as inpatient procedures with burr hole trepanation, and placement of a draining tube which was removed a few days after the surgery depending on the resolution of the patients’ symptoms and the postoperative CT scan. Antithrombotic medication was stopped preoperatively unless the patient’s clinical state required urgent intervention. In the latter case, coagulation was optimized preoperatively, if appropriate. Statin medication remained unchanged. Repeat surgery was performed if the symptoms had not resolved, if the postoperative CT examination showed no sufficient hematoma resolution, if significant hematoma recurrence occurred after initial resolution, or if a wound healing disturbance was observed which did not resolve spontaneously.

The patients’ electronic files were researched for patient characteristics, including age and sex, hematoma site, Markwalder grading score [10] (MGS, Table 1), statin medication at the time of CSDH treatment, antithrombotic medication, repeat surgeries with the causes thereof, other medical conditions (which were used to calculate the Charlson comorbidity index, CCI), and surgical complications including wound healing disturbance and local infection. Additionally, we contacted patients or their relatives via phone and/or by post to obtain informed consent for study inclusion. Missing information was complemented, particularly about possible repeat surgeries at other institutions. Patients lost to follow-up were excluded from the analysis.

**Table 1:** Markwalder grading score

Score	Neurological status
0	Patient neurologically normal
1	Alert and oriented; mild symptoms such as headache; absent or mild neurological deficit, such as reflex asymmetry
2	Drowsy or disoriented with variable neurological deficit, such as hemiparesis
3	Stuporous, but responding appropriately to noxious stimuli; severe focal signs such as hemiplegia
4	Comatose with absent motor responses to painful stimuli; decerebrate or decorticate posturing

Evacuation of bilateral CSDH during the same surgery was considered one procedure. If a patient had surgery for a contralateral CSDH at a later time, only the first surgery was considered. If several repeat surgeries were necessary, only the first was counted.

We then separated the patients into two groups: those who took statins as part of their usual medication (statin group) and those who did not (control group). The rate of repeat surgery for any reason was analyzed as the primary endpoint. Secondary endpoints included repeat surgery for residual hematoma, repeat surgery for recurrent hematoma, and neurological condition as measured with the modified Rankin scale (mRS).

Statistical analysis was performed using JASP 0.14.1.0. We considered the samples normally distributed as per the central limit theorem and used the two-sided *t*-test with Welch correction for parametric data, the Mann Whitney *U* test for ordinal data, and the chi-squared test for categorical data. Finally, we performed a binary logistic regression analysis to account for possible confounding variables. The regression model was built considering age as a covariate and statin use, antithrombotic medication, CCI score, and MGS as factors. The CCI and MGS scores were categorized (CCI 0–1 vs. CCI > 1; MGS 0–1 vs. MGS > 1) for the regression analysis. The model was then developed by backward elimination. We considered the results as statistically significant at a *p* value of < 0.05.

Upon completion of the data collection and calculation of the results, we performed a post hoc power analysis for replication of the numbers Tang et al. reported [20]. At an alpha of 0.05, the analysis yielded a power of 88%.

**Fig. 1:**
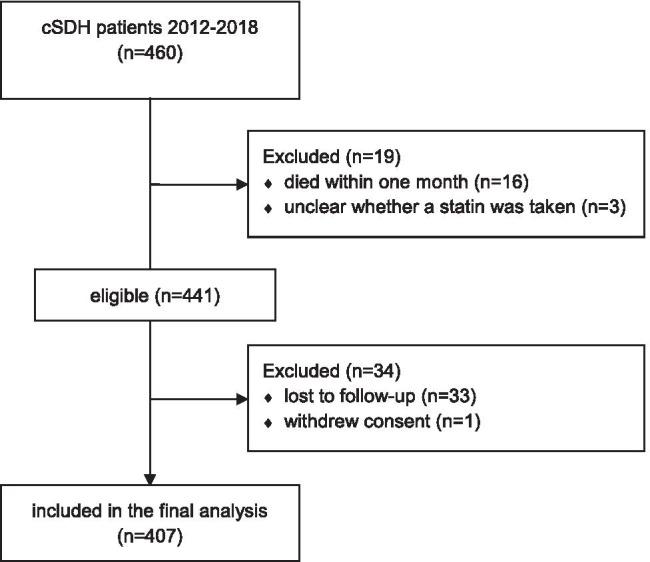
Flowchart of patient inclusion and exclusion

## Results

We identified 460 patients who had undergone burr hole craniotomy for CSDH. None of the patients underwent open craniotomy or twist-drill craniotomy for primary hematoma evacuation. Four hundred seven patients were included in the final analysis (Fig. 1). Of those, 123 took statins while 284 did not. For demographical data, see Table 2. The mean follow-up time was 32.7 ± 16.1 months (control group: 32.9 ± 16.1; statin group 32.1 ± 16.1). None of the patients reported having received repeat surgery at another institution. For the 407 patients included, there was no missing data.

**Table 2:** Patient demographics

	Control group	Statin group	*p*
*n*	284	123	
Male	187 (65.8%)	93 (75.6%)	0.051
Mean age (years)	74.7 ± 13.2	76.6 ± 8.2	0.07
Bilateral hematoma	61 (21.5%)	29 (23.6%)	0.64
Median CCI	1 (IQR = 2)	2 (IQR = 2)	< 0.001
CCI 0–1	176 (62.0%)	59 (48.0%)	0.009
Median MGS	1 (IQR = 1)	1 (IQR = 1)	0.96
MGS 0–1	177 (62.3%)	77 (62.6%)	0.96
Antithrombotic medication	111 (39.1%)	98 (79.7%)	< 0.001

*CCI*, Charlson comorbidity index; *MGS*, Markwalder grading score

A total of 83 patients (20.4%) required repeat surgery. Fifty-seven out of 284 of the control group underwent a second operation (20.1%) while 26 out of 123 of the statin group did (21.1%). Incomplete resolution of the hematoma was the cause of repeat surgery in 27 patients in the control group (9.5%) and 14 patients of the statin group (11.4%), whereas recurrent hematoma caused another surgery in 22 patients (7.7%) and nine patients (7.3%), respectively. Other reasons for repeat surgery were acute postoperative hematoma (seven in the control group and three in the statin group) and one wound healing disturbance in the control group.

Fifty-seven of 280 men required repeat surgery (20.4%) while 26 of 127 women did (20.5%).

Out of 209 patients who received antithrombotic medication, 50 required repeat surgery (23.9%): 25 due to incomplete resolution of the hematoma (12.0%), 18 due to hematoma recurrence (8.6%), and seven due to acute postoperative hematoma (3.3%). Patients not on antithrombotic medication (197) required repeat surgery in 32 cases (16.2%): 15 due to residual hematoma (7.6%), 13 due to hematoma recurrence (6.6%), three due to acute hematoma (1.5%), and one due to a wound healing disturbance (0.51%).

Two hundred thirty-five patients had a CCI score of 0–1 at the time of CSDH evacuation. Forty-three (18.3%) of them required repeat surgery: 20 due to incomplete resolution (8.5%), 19 due to recurrence (8.1%), three due to acute hematoma (1.3%), and one due to a wound healing disturbance (0.43%). Among the 172 patients with a CCI score > 1, 40 had a repeat surgery (23.2%). Twenty-one had a residual hematoma (12.2%), 12 a recurrent hematoma (7.0%), and seven an acute postoperative hematoma (4.1%).

A total of 254 patients presented with an MGS score of 0–1. Fifty-one of them needed a second procedure (20.1%): 23 due to residual hematoma (9.1%), 22 due to recurrent hematoma (8.7%), five due to acute postoperative hematoma (2.0%), and one due to a wound healing disturbance (0.39%). One hundred fifty-three patients had an MGS score > 1. 32 of them had a revision surgery: 18 due to residual hematoma (11.8%), nine due to recurrence (5.9%), and five due to acute hematoma (3.3%).

At the last follow-up, the median mRS was 2.5 (IQR = 6) for patients in the control group and 3 (IQR = 5) for patients in the statin group.

### Primary outcome parameter

There was no significant association between statin use and the frequency of repeat surgery in the chi-squared test (*p* = 0.81).

Upon binary logistic regression, none of the included variables showed a statistically significant association with repeat surgery. After five steps of backward elimination, antithrombotic medication was the last remaining parameter and yielded an alpha error of *p* = 0.07.

### Secondary outcome parameters

Upon logistic regression, there was no significant association between any of the covariates and factors included in this study with the frequency of residual or recurrent hematoma or acute postoperative hematoma. The difference in the neurological status at the last follow-up as measured by the mRS was not significant (*p* = 0.27). Table 3 summarizes the essential results of the study.

**Table 3:** Results

	Control group	Statin group	*p*
Repeat surgery	57 (20.1%)	26 (21.1%)	0.81
Residual hematoma	27 (9.5%)	14 (11.4%)	0.56
Recurrent hematoma	22 (7.7%)	9 (7.3%)	0.88
Acute postop. hematoma	7 (2.5%)	3 (2.4%)	0.99
Wound healing disturbance	1 (0.35%)	0	0.51

## Discussion

We conducted a study in patients who were treated with statins and had to undergo surgery for CSDH. We compared the frequency of repeat surgeries with patients not treated with statins. No reduction in the total rate of repeat surgery was shown. Likewise, no significant difference could be confirmed when separately considering revision procedures due to incomplete hematoma resolution or due to hematoma recurrence.

Statins have long been considered to have beneficial effects beyond lipid lowering [13]. Within the context of CSDH, atorvastatin, in particular, was investigated in numerous studies and was repeatedly found to improve the clinical course. The published articles ranged from small case series [1, 5] to larger studies [6, 20, 25] and two randomized controlled trials (RCT) [7, 22]. Notably, two positive studies were later retracted [9, 29].

Jiang et al. conducted an RCT in which 98 patients with CSDH received 20 mg of atorvastatin and 98 a placebo for 8 weeks. After this period, the hematoma volume reduction was significantly more pronounced in the intervention group. As secondary outcome parameters, patients in the statin group had better neurological function and underwent salvage surgery less frequently [7]. However, the authors excluded patients, among others, who had a history of bleeding or thrombosis, who had cancer, who were taking antiplatelet medication, who had an MGS score ≥ 3, who required surgery, who had been taking statins “for a long time” before the trial or if “for any reason, the researchers believe[d] that the case [was] not suitable for inclusion.” As patients with CSDH frequently have comorbidities and take antithrombotic medication, this precluded patients most likely to develop a CSDH from being studied and prohibits the results of the trial from being generalized to the CSDH population in clinical practice. Furthermore, while 21% of the patients randomized to receive atorvastatin had bilateral hematomas, 38% of the placebo group did. As the bilateral occurrence of CSDH is an independent risk factor for recurrence [12], this constitutes a significant selection bias.

Tang et al. retrospectively studied 245 patients who received burr hole craniotomy for CSDH, 52 among whom had received 20 mg of atorvastatin preoperatively [20]. The authors report a recurrence rate of 15% in the control group and 4.8% in the atorvastatin group within six months. As the decision to administer atorvastatin was made individually by the treating physician, it is difficult to interpret the results.

Okano et al. researched risk factors for postoperative CSDH recurrence in a Japanese cohort and found no significant association with statin therapy [12]. It has to be noted that only 25 of the 448 patients took statins, so the analysis is likely underpowered.

We included 407 patients, 123 of whom were treated with statins. We strived toward meticulous follow-up with little missing data. Upon post hoc power analysis, our study had a power of 88% for the detection of the results Tang et al. had reported. Furthermore, we strived for an accurate description of the reasons for repeat surgery when we distinguished insufficient resolution of the hematoma after its evacuation from recurrent hematoma. This distinction is not commonly made in the literature, although the problem of postoperative residual hematoma due to compartmentalization is well known in day-to-day neurosurgical practice and differs from recurrent CSDH, which is a secondary expansion of a previously reduced hematoma.

Mechanisms of statins that are discussed as potentially promoting pleiotropic effects in CSDH include the inhibition of inflammation, promotion of blood vessel formation and maturation, an elevation of the number of endothelial precursor cells, and the suppression of vascular endothelial growth factor [27]. Preclinical studies suggest a biphasic effect of statins, whereby the aforementioned responses only occur with lower concentrations, while higher statin doses lead to an inhibition both of angiogenesis and of the growth and migration of endothelial precursor cells [21, 23]. The common dosing of atorvastatin in the treatment of CSDH was 20 mg, as described in the studies mentioned earlier. In our study, only 17 patients took atorvastatin, whereas simvastatin was used far more often (Table 4).

**Table 4:** Statin types and dosages

Statin type	No repeat surgery	Repeat surgery	Total
Atorvastatin 10 mg	3	0	3
Atorvastatin 20 mg	4	1	5
Atorvastatin 40 mg	7	2	9
Fluvastatin 40 mg	3	0	3
Fluvastatin 80 mg	3	0	3
Pravastatin 10 mg	1	0	1
Pravastatin 20 mg	2	0	2
Pravastatin 30 mg	1	0	1
Pravastatin 40 mg	1	0	1
Simvastatin 5 mg	1	0	1
Simvastatin 10 mg	6	1	7
Simvastatin 20 mg	30	12	42
Simvastatin 30 mg	1	1	2
Simvastatin 40 mg	34	8	42
Simvastatin 80 mg	0	1	1
None	227	57	284

The dosing of simvastatin needs to be doubled to achieve therapeutic equivalence to atorvastatin [24]. Therefore, 40 mg simvastatin is equivalent to 20 mg atorvastatin. Ninety-five of the 123 patients who were treated with statins in our study took simvastatin, all but one 40 mg or less. Among the patients who took other statins, none surpassed 20 mg atorvastatin equivalence. Thus, we estimate that the pleiotropic effects observed with lower statin concentrations can be assumed in our cohort.

All studies showing a positive effect of statins upon the clinical course of CSDH were conducted in China. It is unknown whether statin effects upon CSDH differ between ethnic groups. Research on the influence of ethnicity upon the pharmacokinetics and pharmacodynamics of statins has shown controversial results with some investigations reporting differences between the Western population and Asians [4, 8, 11] while others failed to detect variance [2, 26]. There is a large number of studies, however, that suggest a lower dosing of statins required in Asians to achieve the same lipid-lowering effects as compared to Westerners.

Our study is limited by its open design with retrospective inclusion of patients which does not allow for randomization. Consequently, patients in the statin group had a higher median CCI score (2 vs. 1) and a larger percentage of CCI ≥ 1 upon categorization (52.0% vs. 38.0%, *p* = 0.009). However, we found no evidence of a higher CCI score being associated with a higher frequency of repeat surgeries. Patients in the statin group were significantly more likely to receive antithrombotic medication (79.7% vs. 39.1%, *p* < 0.001). While antithrombotic medication failed to achieve statistical significance for association with repeat surgery (*p* = 0.07), the study was not designed to detect such a difference. Therefore, the lack of statistical significance needs to be interpreted with caution and the considerably larger fraction of patients taking antithrombotic medication in the statin group might constitute a bias. Only 25 patients took statins, but no antithrombotic medication; two of them required repeat surgery due to incomplete hematoma resolution. This number of patients is too small to be interpreted. Hypothetically, CSDH patients who do not require antithrombotic medication may be a subgroup in which statins may indeed improve the outcome.

Furthermore, we had no standard regimen according to which statins were administered to patients. Instead, we included patients who took statins as part of their daily medication. This has the downside of a significant variation regarding types and doses of statins in contrast to previous studies that strictly investigated 20 mg of atorvastatin in CSDH patients. On the other hand, our patients received statins not only after a CSDH had been diagnosed but already during its development. Therefore, any positive effects could already occur from the very beginning of CSDH formation. Furthermore, our results offer a high degree of external validity since our cohort is representative of the patient population suffering from CSDH in daily practice.

Given the encouraging results of other studies and the generally favorable safety profile of statins[15], it is important to address in future studies whether there is a differential effect of atorvastatin on the outcome in CSDH patients compared to other statins and whether a certain subgroup of CSDH patients—possibly those not requiring antithrombotic medication—is more likely to benefit from statins than others.

Our results do not support the hypothesis that statins reduce the rate of revision surgery after CSDH drain. Therefore, statins are not warranted in patients undergoing surgery for CSDH beyond their well-documented benefits as lipid-lowering drugs and for secondary prevention of cardiovascular disease. Future studies should determine whether atorvastatin has a differential effect on CSDH compared to other statins and whether certain subgroups among CSDH patients may benefit from statin therapy more than others.
